# Best practices in use of research evidence to inform health decisions

**DOI:** 10.1186/1478-4505-4-11

**Published:** 2006-11-20

**Authors:** Judith A Whitworth

**Affiliations:** 1 Chair, WHO Advisory Committee on Health Research, Director, The John Curtin, School of Medical Research, Deputy Director, Menzies Centre for Health Policy, The Australian National University, Canberra ACT 0200, Australia

## Abstract

The WHO Advisory Committee on Health Research (ACHR) is committed to the notion that WHO should exemplify best practice in use of research evidence to inform decisions about health. A major ongoing initiative of the ACHR is the Sub-committee on the Use of Research Evidence (SURE). This group is examining WHOs roles and responsibilities in the use of health research to inform decisions about health. WHOs leadership has expressed strong support for this initiative. The series of articles being published in Health Research Policy and Systems, which examine the methods used by WHO and other organisations to formulate recommendations about health, is part of the background documentation SURE has produced to inform ACHRs advice to WHO.

It is critical that health policy makers look to research, not ignorance, as the basis for action in health, and that health professionals look to evidence, not opinion, as the basis for delivery of care.

## Editorial

WHO is unquestionably the world's leading public health agency. Accordingly its recommendations and actions should be informed by the best available research evidence. Over the last 50 years WHO has had notable successes, but the environment is changing. Increasingly governments, health professionals and consumers are demanding more rigorous processes to ensure that health decisions are well informed, with systematic and transparent processes for synthesis and interpretation of evidence, rather than traditional approaches using expert opinion. WHO has the mandate to capitalise on these advances and to play a leadership role with member states.

The WHO Advisory Committee on Health Research (ACHR) is committed to the notion that WHO should exemplify best practice in use of research evidence to inform decisions about health. A major ongoing initiative of the ACHR is the Sub-committee on the Use of Research Evidence (SURE). This group is examining WHO's roles and responsibilities in the use of health research to inform decisions about health. WHO's leadership has expressed strong support for this initiative. The series of articles being published in Health Research Policy and Systems, which examine the methods used by WHO and other organisations to formulate recommendations about health, is part of the background documentation SURE has produced to inform ACHR's advice to WHO.

ACHR looks forward to an ongoing role in promotion of best use of evidence in WHO's policies, recommendations and guidelines. These are essential for WHO to maintain its leadership role as the premier international health organisation in quality of advice based on best research evidence, consistent both with international best practice and WHO's key normative role as a standard setter.

An article on EVIPNet in the Lancet recently pointed out that policy makers often see research as the opposite of action, rather than as the opposite of ignorance [1]. Only this week I heard a senior public health officer bemoan the fact that decision makers preferred policy-based evidence to evidence-based policy. Realistically, policy will be informed by, rather than based on, evidence, because so many other factors eg feasibility, equity, politics enter the equation. Similarly health professionals use evidence not in isolation, but in the context of individual patient characteristics and preferences.

It is critical that health policy makers look to research, not ignorance, as the basis for action in health, and that health professionals look to evidence, not opinion, as the basis for delivery of care.
